# Impact of chlorhexidine and emollient cleansing on bloodstream infections in a Neonatal ICU

**DOI:** 10.4102/sajid.v40i1.718

**Published:** 2025-09-20

**Authors:** Pervashni Padayachee, Fathima Naby, Partson Tinarwo, Sumayya Haffejee, Trudy Martin, Vikash Deonundhan, Yashika Umichand, Moherndran Archary

**Affiliations:** 1 Department of Paediatrics and Child Health, Faculty of Health Sciences, University of Kwazulu-Natal, Nelson R. Mandela School of Medicine, Durban, South Africa; 2 Department of Paediatrics and Child Health, Pietermaritzburg Metropolitan Hospital Complex, Pietermaritzburg, South Africa; 3 Department of Biostatistics, Faculty of Health Sciences, University of KwaZulu-Natal, Nelson R. Mandela School of Medicine, Durban, South Africa; 4 Department of Medical Microbiology, Faculty of Health Sciences, University of KwaZulu-Natal, Nelson R. Mandela School of Medicine, Durban, South Africa; 5 National Health Laboratory Service, Pietermaritzburg Metropolitan Hospital Complex, Pietermaritzburg, South Africa; 6 Discipline of Pharmaceutical Sciences, Faculty of Health Sciences, University of KwaZulu-Natal, Durban, South Africa; 7 Department of Pharmacy, Pietermaritzburg Metropolitan Hospital Complex, Pietermaritzburg, South Africa; 8 Natal College of Nursing, Greys Hospital Campus, Pietermaritzburg, South Africa; 9 Natal College of Nursing, Victoria Mxenge Hospital Campus, Durban, South Africa; 10 Department of Paediatrics, Victoria Mxenge Hospital, Durban, South Africa

**Keywords:** neonatal, sepsis, chlorhexidine, emollient, mortality, blood stream infections

## Abstract

**Background:**

Among hospital-born babies, infections account for 4% – 56% of deaths in the neonatal period, with bloodstream infections being up to 20 times more frequent in low- and middle-income countries (LMICs). The cleansing of infants with chlorhexidine (without emollient) has been the standard of care in Grey’s Hospital Neonatal Intensive Care Unit (NICU) for over a decade.

**Objectives:**

This study evaluated the impact of adding an emollient to chlorhexidine on the prevalence of microbiologically confirmed infections.

**Method:**

A retrospective observational cohort study of all microbiologically confirmed bloodstream infections in the neonatal unit before (01 June 2022 to 30 November 2022) and after (01 December 2022 to 31 May 2023) the implementation of the quality improvement project was conducted. The rates of microbiologically confirmed infections were compared between the two periods.

**Results:**

There was a total number of 2741 positive blood cultures over the study period with a significant reduction in positive cultures in the intervention group (46.1% vs. 27%, *p* < 0.001) but no significant reduction in the proportion of different organisms (Gram-positive, Gram-negative, and fungal isolates) in the two groups (*p* = 0.58) Gram-positive organisms accounted for most infections in the pre- and post-intervention study groups. There was no significant reduction in the rate of sepsis-associated mortality (*p* = 0.20).

**Conclusion:**

Further research evaluating the response in larger study populations is needed.

**Contribution:**

Although there was no significant reduction in sepsis-associated mortality, cleansing infants using chlorhexidine with an emollient may reduce bloodstream infections in this vulnerable population in LMICs.

## Introduction

The Global Burden of Disease (GBD) survey estimates 1.3–3.9 million new neonatal sepsis cases, which contribute to approximately 203 000 avoidable deaths annually. Premature and low-birth-weight (LBW) infants have a higher risk of sepsis, with a mortality rate of 17.6%. Seventy-five per cent of all neonatal deaths occur in low- and middle-income countries (LMICs), especially in South-East Asia and sub-Saharan Africa. Therefore, it is crucial to identify a feasible, simple, and cost-effective means of reducing sepsis in hospitalised infants in LMICs.^1,2,3,4^

Bloodstream infections are the most common nosocomial infection in neonates and occur up to 20 times more frequently in LMICs than in high-income countries (HICs).^5^ Poor infection prevention and control due to lack of trained staff and basic resources, overcrowding, faulty equipment, and rising antimicrobial resistance in LMICs contribute to the higher burden of sepsis-related incidence and mortality.^1,5^ Gram-negative organisms, a common cause of nosocomial and community-acquired neonatal bloodstream infections, are a major obstacle in the global fight to improve child survival.^6^ Bacteraemia is usually preceded by colonisation in hospitalised neonates. This finding has been demonstrated for a broad range of neonatal pathogens, including Gram-negative and Gram-positive bacteria.^6^

The skin is an important entry point for neonatal pathogens.^7^ Studies in developing countries indicate that cleansing of newborn skin with chlorhexidine decreases neonatal mortality. Chlorhexidine is a topical antiseptic with a broad spectrum of activity against Gram-positive and Gram-negative bacteria, facultative anaerobes, yeasts, and viruses, including HIV, thereby reducing skin colonisation.^7,8^ Numerous studies have demonstrated the safety of chlorhexidine in neonates. It is an inexpensive and simple practice with the potential to decrease sepsis-associated morbidity and mortality in low-resource settings.^3^

The addition of an emollient to chlorhexidine solution may lower the risk of invasive bacterial infection by improving skin integrity via the incorporation of lipids.^9^ Recent evidence suggests that emollient therapy enhances postnatal growth, decreases hospital-acquired infection, and may both reduce mortality and improve neurodevelopmental outcomes.^10,11,12,13,14,15,16^ The World Health Organization (WHO), after recently evaluating evidence, introduced a conditional recommendation to use emollients in premature and LBW neonates. They also request additional research, especially in Africa.^10,17,18^

While interventions such as staff education, hand hygiene, and environmental cleaning are well-established universal components of infection control, there are few published studies from hospitals in LMICs on using chlorhexidine cleansing to prevent sepsis and associated deaths.^5^ Furthermore, while chlorhexidine cleansing of babies is standard practice in some neonatal units, including the unit where the study was conducted, there are few studies evaluating the use of chlorhexidine-containing emollient and its effects on infant sepsis.

This study was therefore conducted to determine whether cleansing infants with chlorhexidine-containing emollient helps reduce the rate of bloodstream infections and mortalities in the neonatal unit in a South African tertiary hospital.

## Research methods and design

### Study location and design

A retrospective observational cohort study evaluating the impact of cleansing with chlorhexidine-containing emollient on all microbiologically confirmed infections in babies admitted to a tertiary neonatal unit at Grey’s Hospital between 01 June 2022 and 31 May 2023 was conducted. The study site was the neonatal unit at Grey’s Hospital, a public tertiary hospital in KwaZulu-Natal, South Africa. The hospital serves the west of KwaZulu-Natal and is the referral centre for 18 other hospitals. The neonatal unit has 24 beds and is equipped with invasive and non-invasive ventilators and continuous positive airway pressure machines for critically ill neonates. It is one of only two hospitals in KwaZulu-Natal able to perform neonatal surgery.

Cleansing neonates with chlorhexidine (without emollient) has been the standard of care in Grey’s Hospital NICU for over a decade. The addition of an emollient, glycerine, to the chlorhexidine was implemented on the 01 of December 2022 as part of a quality improvement project due to the significant increase in resistant organisms cultured from severely ill and stable neonates in the unit. To constitute the cleansing solution, 100 mL of glycerine and 100 mL of chlorhexidine were added to 4800 mL of sterile water to make a 2% solution, decanted into 100 mL squeeze bottles and placed at each patient’s bedside. This study evaluated the impact of this change in practice on the prevalence of microbiologically confirmed infections. We compared the mortality and the rates of all microbiologically confirmed infections in the neonatal unit before and after the implementation of the quality improvement project. The rates of microbiologically confirmed infections 6 months prior to the intervention (neonates who were cleaned with chlorhexidine without emollient from 01 June 2022 to 30 November 2022) were compared to the sepsis rates over the intervention period of 6 months (neonates who were cleaned with chlorhexidine containing emollient from 01 December 2022 to 31 May 2023).

### Study population

All neonates admitted in the NICU (neonatal unit) were cleaned with chlorhexidine (sparing the head and face) daily or twice daily if considered to be clinically septic, excluding those with severe skin disease, open wounds, birth weight < 1.5 kg or gestational age < 32 weeks.

A clean gauze pad was moistened with cleansing solution and applied to babies from the jawline down, after which the skin was allowed to air dry. In the 6 months before the intervention (from June 2022 to November 2022), the babies were cleaned with the 2% chlorhexidine solution *without* emollient. During the intervention over the next 6 months (from December 2022 to May 2023), infants were cleaned with the 2% chlorhexidine solution *with* emollient.

### Sample collection

Blood cultures were collected on all babies on admission and repeated on patients with suspected new-onset sepsis (as suggested by clinical features or blood results) as per the standard of care in the unit. Paediatric blood culture bottles were inoculated with 0.5–1 mL of blood and incubated in the BacT/Alert Blood culture instrument. Blood cultures were incubated for 5 days at 37 °C. The blood cultures were continuously monitored for growth by the instrument. If micro-organisms were present in the sample, carbon dioxide was produced as micro-organisms metabolised the substrates in the culture medium. Those generating sufficient carbon dioxide for detection by a light sensor were considered positive.

Aliquots from the positive cultures were subcultured onto chocolate agar, 5% blood agar, and MacConkey agar plates. Organisms were identified based on Gram staining, colony morphology, and biochemical features using the Vitek MS Prime and Vitek 2 instruments. Antibiotic susceptibility was determined primarily with the Vitek 2 instrument, which uses broth dilution. For a small proportion of the organisms, the Kirby-Bauer disc diffusion method was used.

### Data analysis

Data were captured onto Microsoft Excel sheets, and statistical analysis was done with *R* Studio software version 4.4.2. Data were summarised using descriptive statistics. Categorical data were summarised using frequencies and percentages. The frequency distribution of numeric data was analysed for normality, and means or medians were used accordingly. Chi-square tests and *t*-tests/Wilcoxon rank-sum tests were used for categorical data and numerical data, respectively. *p* < 0.05 was considered statistically significant.

### Ethical clearance

An ethical clearance certificate was obtained from the Biomedical Research Ethics Committee at the University of KwaZulu-Natal, South Africa (approval number: BREC/00005331/1023). A waiver of informed consent from the parents or guardians was granted, as the study was a retrospective evaluation of clinical practice. Approval was obtained from the KZN Department of Health and the Grey’s Hospital management.

## Results

### Population characteristics

All blood cultures in the pre-intervention period (chlorhexidine baths without emollient; *N* = 1617) and intervention period (chlorhexidine baths with emollient; *N* = 1124) were included in the study. A total of 2741 samples were analysed during the study period, with 1049 positive and 1692 negative cultures (Table 1). The number of admissions to the neonatal unit was similar in the pre-intervention and intervention periods (298 vs. 299, *p* = 0.95). There were 1617 blood cultures performed in the pre-intervention period and 1124 cultures performed in the intervention period on infants who met the inclusion criteria. There were more male infants in both periods (45.5% vs. 51%; *p*-value 0.014; Table 1).

**TABLE 1:** Population characteristics and culture results of neonates in the pre-intervention (*N* =1617) and intervention (*N* = 1124) periods.

Variable	Pre-intervention (*N* = 1617)					Intervention (*N* = 1124)					Overall (*N* = 2741)					*p* †
	*n*	%	Median	IQR	Min–Max	*n*	%	Median	IQR	Min–Max	*n*	%	Median	IQR	Min–Max	
**Gender**																0.017
Female	673	41.6	-	-	-	424	37.7	-	-	-	1097	40.0	-	-	-	0.130
Male	735	45.5	-	-	-	573	51.0	-	-	-	1308	47.7	-	-	-	0.014
Unknown	209	12.9	-	-	-	127	11.3	-	-	-	336	12.3	-	-	-	0.642
**Age (days)**	1583	-	17.0	6.00–42.0	0–29 200	1118	-	15.5	4.00–38.0	−1.00–7310	2701	-	16.0	5.00–41.0	−1.00–29 200	0.012‡
**Age (days)**																0.005
0–7	296	18.3	-	-	-	220	19.6	-	-	-	516	18.8	-	-	-	1.000
8–14	252	15.6	-	-	-	142	12.6	-			394	14.4	-	-	-	0.247
15–21	139	8.6	-	-	-	91	8.1	-	-	-	230	8.4	-	-	-	1.000
21–28	118	7.3	-	-	-	96	8.5	-	-	-	214	7.8	-	-	-	1.000
> 28	697	43.1	-	-	-	518	46.1	-	-	-	1215	44.3	-	-	-	1.000
Unknown (missing)	34	2.1	-	-	-	6	0.5	-	-	-	40	1.5	-	-	-	0.004
Unknown (negative)	0	0.0	-	-	-	1	0.1	-	-	-	1	0.0	-	-	-	1.000
Unknown (> 6 months)	81	5.0	-	-	-	50	4.4	-	-	-	131	4.8	-	-	-	1.000
**Blood culture result**																0.001
Negative	871	53.9	-	-	-	821	73.0	-	-	-	1692	61.7	-	-	-	< 0.001
Positive	746	46.1	-	-	-	303	27.0	-	-	-	1049	38.3	-	-	-	< 0.001
**Classification**																0.582
Gram-negative	152	20.4	-	-	-	59	19.5	-	-	-	211	20.1	-	-	-	-
Gram-positive	528	70.8	-	-	-	211	69.6	-	-	-	739	70.4	-	-	-	-
Fungal	66	8.8	-	-	-	33	10.9	-	-	-	99	9.4	-	-	-	-

IQR, interquartile range (Q1–Q3); Min, minimum; Max, maximum.

† , Chi-square test; ‡, Mann-Whitney U test.

### Blood culture results

**FIGURE 1:**
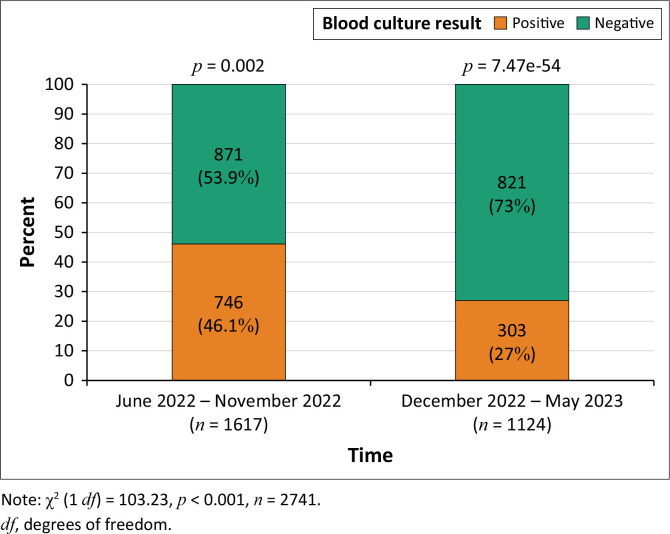
Negative and positive blood cultures in pre-intervention and intervention periods

There were 746 positive cultures out of 1617 (46.1%) cultures performed in the pre-intervention period and 303 positive cultures out of 1124 (27%) during the intervention period (Figure 1). Positive cultures were significantly lower in the post-intervention period (46.1% vs. 27%, *p* < 0.001).

### Culture-proven sepsis

**FIGURE 2:**
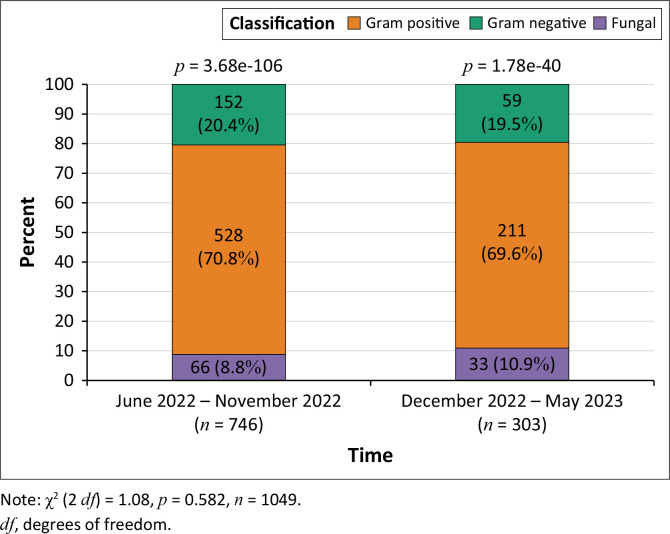
Classification of positive blood culture results in the pre-intervention and intervention periods.

There was no significant difference in the proportion of different organisms in the pre-intervention and intervention groups (746 vs 303, *p*-value 0.582). Gram-positive organisms constituted most of the isolates in both groups, followed by gram-negative and fungal organisms (Figure 2).

### Isolated organisms

**FIGURE 3:**
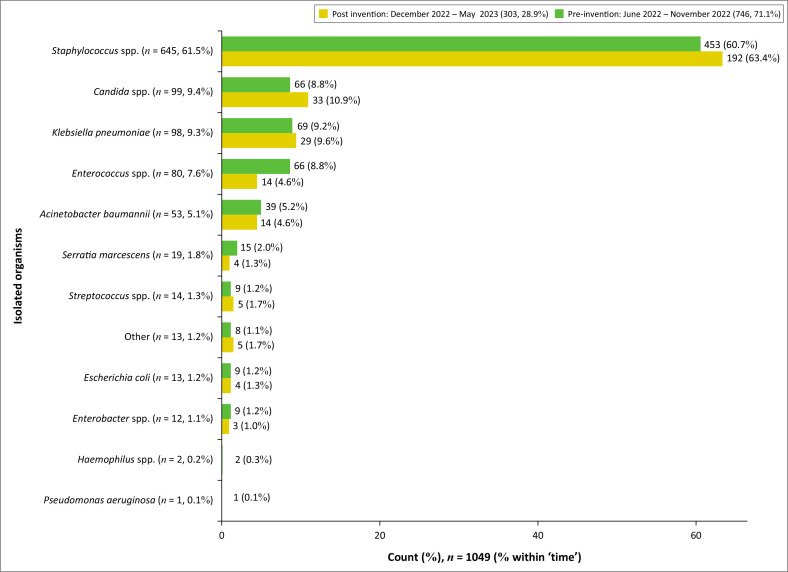
Percentages of isolated organisms in pre-intervention and intervention periods (*n* = 1049).

The most isolated organism in both periods was *Staphylococcus* spp. (63.4% and 64.7%), followed by *Candida* spp., *Klebsiella pneumoniae, Enterococcus* spp., *Acinetobacter baumaunii, Serratia marcescens, Streptococcus* spp., *Escherichia coli, Enterobacter* spp., *Haemophilus* spp., and *Pseudomonas aeruginosa* (Figure 3).

There was an increase in the prevalence of *Staphylococcus* spp. (from 60.7% to 63.4%), *Candida* spp. (from 8.8% to 10.9%), *K. pneumoniae* (from 9.2% to 9.6%), *Streptococcus* spp. (from 1.2% to 1.7%), *E. coli* (from 1.2% to 1.3%), and other organisms (from 1.1% to 1.7%) in the intervention group (*p*-value 0.60). A reduction was noted in *Enterococcus* spp. (from 8.8% to 4.6%), *A. baumaunii* (from 5.2% to 4.65%), *S. marcescens* (from 2% to 1.3%), and *Enterobacter* spp. (from 1.2% to 1%; *p*-value 0.60; Figure 3).

### Mortality and patient characteristics

Deaths were similar: 30 deaths of the 298 admissions (10%) in the pre-intervention period versus 39 deaths of 299 admissions (13%) in the intervention period (*p*-value 0.25). HIV-unexposed infants constituted more than half of the mortalities in both groups (22/30 [73.3%]; 24/39 [61.5%], *p*-value 0.45). Sepsis was the most common cause of death (12/30 [40%]) in the pre-intervention group and the second most common cause (10/39 [25.6%]) in the intervention group, with a decline of 14.4% in the sepsis associated-mortality rate in the intervention period (*p*-value 0.20).

## Discussion

Several studies have demonstrated that chlorhexidine cleansing decreases skin colonisation, and this action combined with the protective effect of emollient on the skin barrier can potentially lower the risk of invasive bloodstream infections, a common cause of infant morbidity and mortality.^2,3^ Some studies evaluated chlorhexidine cleansing and emollient application separately to prevent sepsis and death. Two local studies have evaluated the effect of emollient application immediately after chlorhexidine baths on skin colonisation.^9,19^ Few studies have evaluated the effect of combining the two agents in one preparation.

Our study documented a significant reduction of 19.1% in positive blood cultures when cleansing with chlorhexidine-containing emollient compared to cleansing with chlorhexidine alone (Figure 1). Notably admissions in both periods were similar (298; 299) but with significantly fewer blood cultures collected in the intervention period (1617;1124), likely because of a decreased incidence of congenital and hospital-acquired sepsis and a reduced need for repeating blood cultures (Table 1, Figure 1).

There was a 1.2% decline in the number of Gram-positive isolates and a 0.9% decline in the Gram-negative isolates in the intervention group (Figure 1). The commonly isolated bacterial organisms in the pre-intervention and intervention groups were similar to a review of neonatal sepsis in 19 developing countries by Downie et al. in 2013, which showed that over half the cases were attributable to *S. aureus, E. coli,* or *Klebsiella* spp.^20,21^ Our study also illustrated a significant proportion of fungal sepsis, largely *Candida* spp., in both groups of patients, with a slightly higher prevalence in the intervention group (8.8%; 10.9%; Figure 2). A more recent systematic review and meta-analysis of the leading pathogens causing neonatal sepsis in developing countries published in 2021 by Zelellw et al. showed that the commonest pathogens causing hospital-acquired sepsis were *Klebsiella, S. aureus, E. coli*, coagulase-negative *Staphylococci, Pseudomonas* spp., *Enterobacter* spp., and *Candida* spp.^22,23^ In Africa, the most common causes of neonatal sepsis are *S. aureus* (27.8%) and *Klebsiella* spp. (29.8%).^22^

Our study showed an increase in the number of *Staphylococcus* spp., *Candida* spp., *K. pneumoniae, Streptococcus* spp., and *E. coli* in the intervention group, with a reduction noted in the number of *Enterococcus* spp., *A. baumaunii, S. marcescens*, and *Enterobacter* spp., although these differences were not statistically significant.

Mortality increased from 10% to 13% in the intervention period, but with a 14.4% decrease in sepsis-associated mortalities, which was not statistically significant (*p*-value 0.20). Randomised controlled trials have illustrated that cord application of chlorhexidine decreases neonatal mortality in some community settings with high mortality rates and that skin application may result in a similar effect by decreasing skin colonisation, but several reviews evaluating the effects of chlorhexidine on both sepsis and mortality were inconclusive.^19,24,25^ Westling et al. found that chlorhexidine bathing at admission was associated with a reduced risk of bloodstream infections in a single-centre study in Zambia,^6^ and Quach et al. showed a 67% reduction in central line-associated bloodstream infections after bathing babies > 1 kg with 2% chlorhexidine.^26^ The NeoHCG trial published by Russell et al. in 2024 evaluated the safety and efficacy of whole-body chlorhexidine gluconate cleansing with or without sunflower oil as an emollient in hospitals in South Africa and Bangladesh. Investigators suggested that the combination was likely safe and feasible to be implemented in a larger trial. However, the study showed dense and rapid colonisation of skin by bacteria in the first days of life irrespective of chlorhexidine application, and there was no substantial evidence to support its role for reducing sepsis.^19^

We found no adverse events associated with chlorhexidine-containing emollient on the infants. There have been previous reports of hypothermia^3,27,28^ and adverse skin reactions ranging from mild forms like transient erythema to more severe forms, including skin breakdown, when chlorhexidine alone was used on the skin of preterm babies. Investigators suspected that this may have also been attributed to the use of alcoholic preparations of chlorhexidine or the application of occlusive dressings to skin surfaces before adequate drying.^6,29,30,31^

The addition of emollient, which serves as a protective barrier on the skin, may potentially reduce adverse skin reactions. Emollient therapy for neonates is now recommended globally by the WHO because of evidence highlighting its beneficial properties on the skin and neurodevelopment and reduction in neonatal sepsis rates.^10,11,12,13,14,15,16^

Our chlorhexidine-glycerine solution was prepared at a low cost by the hospital pharmacy using locally produced ingredients and without supply issues to the neonatal unit.

There were a few important limitations of the study. The results may have included some false-negative and false-positive cultures because of factors related to poor culture collection technique, including poor aseptic technique and inadequate sample inoculation into blood culture bottles.

Although we did not encounter problems with the production and supply of the chlorhexidine-containing emollient during the study, it is a very likely potential problem in any limited-resource setting when considering more widespread and continuous use of this practice.

The limited data set did not allow for distinction between congenital and hospital-acquired infections. (The POPIA ACT protects patient identification; hence, we could not access patient files to obtain dates of admission, which would have allowed distinction between congenital and hospital-acquired infections). The study design may have contributed to bias in data collection, analysis, and reporting of adverse events.

## Conclusion

In conclusion, we recommend further evaluation of the intervention in a larger multi-site cluster randomised trial. It is also necessary to evaluate whether this practice is associated with chlorhexidine resistance and potential cross-resistance between chlorhexidine and antibiotics.
